# Understanding regional talent attraction and its influencing factors in China: From the perspective of spatiotemporal pattern evolution

**DOI:** 10.1371/journal.pone.0234856

**Published:** 2020-06-18

**Authors:** Beibei Hu, Yingying Liu, Xiaoxiao Zhang, Xianlei Dong

**Affiliations:** School of Business, Shandong Normal University, Jinan, China; Institute of Geographic Sciences and Natural Resources Research (IGSNRR), Chinese Academy of Sciences (CAS), CHINA

## Abstract

Talents are not only an important strategic resource for promoting regional development but also a core element for maintaining competitiveness. We organize the evaluation index system of regional talent attraction into the following four aspects: regional development, industry development, income and regional environment. Combined with the talent possession of 31 provinces (cities) from 2010 to 2018, we establish a regression equation of the relationship between the evaluation index and talent possession by using a stepwise regression and the Bayesian prior function. Simultaneously, we apply the spatial autocorrelation analysis method to measure the correlation and agglomeration degree of the talent attraction level of provinces and municipalities in China. The results reveal the following. (1) From 2010 to 2018, the talent attractiveness level of China's provinces shows a steady upward trend with an average annual growth rate of 5.804%. The regional environment has the highest score, and the income level has the lowest score. (2) The level of talent attraction in China shows a decreasing trend from east to west, and the ranking in 2018 was "East Coast > North Coast > Southern Coast > Middle Yangtze River > Middle Yellow River > Southwest > Northeast > Greater Northwest". The trend of spatial agglomeration is apparent and gradually increases over the years. The numbers of hot and cold spots are relatively large and concentrated in the eastern coast and western region, respectively. (3) The level of economic development, quality of people's life, and level of the development of the tertiary industry have a great impact on the attractiveness of talents. Talents also pay more attention to regional medical, education and transportation indicators. These research results can provide some guidance and references for the formulation of talent introduction policies in various provinces and municipalities.

## Introduction

Talent refers to workers who have certain professional knowledge or specialized skills, can carry out creative work, and contribute to society. Their ability and quality in human resources are relatively high. In 1982, relevant documents released by the State Council imposed related restrictions on talents in China. The first type of talent comprises people with professional skills, such as technicians and above, while the second type of talent comprises people with academic qualifications of secondary school or above. This definition has been widely used to date [1]. According to statistics, at the end of 2015, China's human resources reached 175 million, accounting for 15.5% of the total. There are 48.5 R&D personnel per 10,000 labor forces, and the proportion of highly skilled personnel reaches up to 29% [2]. Talents have an important influence on China's economic and social development. With economic development and social progress, talents play a more crucial role in promoting improvement in regional development. The overall layout of the "five in one" note that deepening the structural reform of the supply side requires strengthening the cultivation of human capital and promoting the spirit of craftsmanship and the spirit of model workers. The theory of talent attraction can be divided into the following two levels: talent attraction based on the economy and talent attraction based on the scope of human resource management [1]. Compared with the policy arrangement, talent attraction has intangible and cumulative characteristics and is concentrated in the environmental system, social and cultural atmosphere, and living environment [3,4]. The foundation of the theory of talent mobility was formed during the market economy period. Various models of talent mobility have noted that when the working conditions do not meet the expectations of talents, talents may leave. In modern China, the competition for talents in various regions is becoming increasingly fierce. Talent competitiveness exhibits characteristics with large differences between the east and west, and gradually increases from inland to the coastal areas [5]. All provinces are exerting effort to create conditions conducive to the influx of talents and actively increase attractiveness. It is especially important to formulate policies to meet the needs and increase the introduction of talents.

Different scholars have studied talent attraction in different regions and fields and the influencing factors from the following three perspectives. First, research is conducted by constructing a talent attraction evaluation index system. For example, many scholars employ brainstorming, expert advice or the Delphi method [6] to select a talent attraction evaluation index to construct a talent attraction evaluation index system. Then, the weight of each indicator is calculated, and a talent attractiveness model is built based on the analytic hierarchy process [3], entropy method [7], principal component analysis method [8], and factor analysis method [9]. Finally, the outcomes of talent attractiveness can be determined. In recent years, some new methods in this area have been proposed, such as structural equation modeling [10], combinations of the stochastic forest method, logistic regression model [11] and wrapped feature filtering [12], etc. Second, the attraction of regional talents and the influencing factors have been studied from the perspective of spatial patterns. For example, at the macro level, from the perspective of urban classification, the factors influencing talent attraction in first-tier cities and quantitative analyses of the impact mode and degree of influence have been addressed [13]. The specific research areas mainly include Shanghai, Shenzhen, Henan, Wuhan, Xinjiang and other cities. A previous study found that talent attraction in Shanghai is mainly influenced by the environment, social and cultural atmosphere and living environment [3]. The urban financial environment, cultural atmosphere, living environment and talent policy have a significant impact on the attractiveness of Shenzhen financial talents [14]. The industrial cluster environment has the ability to gather talents [15]. High-speed railways also have a greater impact on the level of talent attraction in surrounding cities [9]. The loss of talent in Xinjiang is mainly due to economic factors [16]. At the micro level, talent attraction in specific fields or enterprises and the influencing factors have been examined. A previous study found that environmental value congruence has a positive impact on attractiveness through organizational reputation [17]. To a science popularization organization, the three factors most relevant to attractiveness are promotion, competence and prestige [18]. A family business identity reduces the attractiveness of businesses to external talent [19]. The conscious implementation of corporate social responsibility can help companies attract talents [20].

Third, from the perspective of talent policy, some studies explore the attraction of regional talents. The formulation and implementation of talent policy mainly involve the government, enterprises and universities, and introduction policies mainly target high-level talents, innovative and entrepreneurial talents and overseas talents. Some studies have noted that the current attractiveness evaluation does not distinguish between objective attractiveness and policy attractiveness but focuses on the overall attractiveness evaluation, which is not conducive to policy improvement. By constructing relevant indicators of policy attractiveness, the factor analysis method is used to measure the objective attractiveness and absolute attractiveness of policies, and the evaluation results of high-level talent attractiveness of provinces at the policy level are obtained [21,22]. Based on the content analysis method, some studies examine the development policy of scientific and technological innovation talents from the perspective of the development process of scientific and technological innovation talents [23, 24, 25]. According to a questionnaire survey concerning the introduction of overseas talents by universities, some research proposes countermeasures and suggestions for improving the market-oriented system and mechanism of introduction of overseas high-level talents in cities from six aspects [26]. Four conceptions regarding talent introduction in universities are discussed under the background of “double first-rate” construction, and “introduction, integration, utilization and retention” are considered the basic links in talent introduction in universities [27]. Although talent introduction policy can attract talents to choose jobs, with the introduction of policies in various regions, a series of problems, such as excessively utilitarian talent recruitment, irrational competition among local governments and the lack of future planning, have been gradually exposed in this “talent competition war” [28]. Concurrently, the convergence of local policies discourages the enthusiasm of talents and increases the risk of the partial waste of talents [29]. Considering the above problems, some studies have indicated that talent introduction should meet development needs, perfect the guarantee system, respect market rules, break the old model of attracting talents only with academic qualifications, and rely on industry attraction [30].

Based on the above research status and development trends, it can be concluded that previous studies concerning evaluation models and the influencing factors of talent attraction have obtained robust research results, although a series of problems remain to be solved and reflected. First, regarding methodology, most research investigating the construction of a talent attraction evaluation index system adopted the method of assigning weight to the index. If the weighting method is too subjective or objective, systematic errors could incur. Second, during the selection of evaluation indicators, most previous studies selected a small number of representative indicators and information coverage that is not comprehensive, which could also have a certain impact on the results. Finally, regarding the spatial pattern of talent attraction and its influencing mechanism, most studies focused on investigating talent attraction in a single region or a single field and rarely analyzed the level of talent attraction in China from an overall perspective. In summary, with the background of strengthening human resource cultivation in China, this paper selects a comprehensive and systematic index, uses the variable selection model and stepwise regression method, studies the influencing factors in China’s provincial talent attraction and its pattern of time and space dynamic evolution, and provides talent policies for various areas to provide guidance and reference.

## Data and index selection

Based on existing research results concerning talent attraction at home and abroad [3,10], this paper mainly measures the attractiveness of Chinese regional talents using four first-level indicators, including regional development, industry development, income and regional environment, as shown in Table 1. According to the systematic, comprehensive, scientific authenticity and operability principle of the indicator system, we choose 24 secondary indicators of regional development, 31 secondary indicators of industrial development, 21 secondary indicators of income and 50 secondary indicators of the regional environment as shown in Tables 1–4 in the S1 Appendix. The research area mainly includes 31 provinces, cities and autonomous regions in China (due to the lack of data, Hong Kong, Macao and Taiwan of China are not included in this study). The data are mainly derived from the website of the National Bureau of Statistics and the statistical yearbook of provinces and cities of China from 2011 to 2019.

**Table 1:** The evaluation indicators for city talent attractiveness.

Primary indicator	Secondary indicators	Number of tertiary indicators
Regional development	National economic accounts	3
	Finance and investment	2
	Foreign economic trade	5
	City overview	6
	People's life	5
	Social security	3
Industry development	Total social investment in fixed assets	19
	Added value in 19 industries	19
	Three major industrial value added indexes	3
Income	The average salary of employees in 19 industries	19
	The average salary of all employees	2
Regional environment	Traffic and security	7
	Greening and pollution	18
	Education and health care	15
	Housing and shopping	10

The selection of index system is an inherently subjective process. However, our purpose was to define an initial list of influencing indicators that could reasonably be expected to yield useful information about changing the attractiveness of Chinese regional talents. Our objective was not to formulate a definitive, exhaustive set of all possible influencing indicators. In our research, referring to previous studies, we built our indicators from the four aspects of regional economy, industries, income and environment. We supplemented the influencing indicator systems from across several decades, to give a total of 126 indicators. Although this has made our model much more complex, the proposed variable selection model below can deal with this issue and pick the valuable variables out automatically. Thus, our strategy is to collect as many influencing indicators as possible and then select the significant ones from the set. Future work may fine-tune this set of influencing indicators to increase the accuracy and validity of the model.

Considering the large gap in the economy, population and geographical area among provinces and cities, using the absolute data of the selected indicators may cause deviations. Therefore, this paper uses each index value divided by the corresponding area to show the development level, such as the number of employees per unit area to show the relative level of employees in each area.

## Methods

### Talent attraction measurement model

Based on the Bayesian prior function and stepwise regression model [31], the functional equation of the relationship between the comprehensive evaluation index and the number of employees is constructed, and the regression coefficient of each evaluation index is used as the weight of the index to calculate the talent attraction level of each province. The specific steps are as follows:

Step 1: Data standardization. We record the matrix of all observations of 126 secondary indicators as *X* = (*x*_*ij*_). Simultaneously, the column vector formed by the number of employees in each province (city) is recorded as (*y*_*i*_) among them, *i* = 1,2,⋯,279; *j* = 1,2,⋯,126. All comprehensive evaluation indicators and number of employees are standardized on a 0–1 range. We obtain the standardized matrices $\left\{x_{ij}^{\prime }|x_{ij}^{\prime }=\frac{x_{ij}-x_{j\min}}{x_{ij}-x_{j\max}}\right\}$ and $\left\{y_{i}^{\prime }|y_{i}^{\prime }=\frac{y_{i}-y_{i\min}}{y_{i}-y_{i\max}}\right\}$, where *x*_*j*min_,*x*_*i*max_ represent the minimum and maximum values of all observed values of the *j*^*th*^ indicator, respectively, and *y*_min_,*y*_max_ represent the minimum and maximum values of the number of employees, respectively.
Step 2: Construction of the variable selection model. This model is based on the Bayesian prior sparse function and stepwise regression model. First, let *β* represent the set of coefficients of independent variables; when *β*_*k*_ = 0, set *γ*_*k*_ = 0; otherwise, set *γ*_*k*_ = 1. Then, *γ*_*n*_ follows the (0–1) Bernoulli distribution, and the probability density function can be written as follows:
  $$f=p_{k}^{\gamma_{k}}{\left(1-p_{k}\right)}^{1-\gamma_{k}}=\{\begin{array}{l} p_{k},\;\gamma_{k}=1\\ 1-p_{k},\;\gamma_{k}=0 \end{array}$$ (1)

Simultaneously, let *γ* = (*γ*_1_,*γ*_2_,…,*γ*_*n*_)^*T*^, and the probability density can be written as follows:

$$\gamma ∼\prod_{k=1}^{n}{\left(1-p_{k}\right)}^{1-\gamma_{k}}$$ (2)

where *p*_*k*_ can be determined according to the ideal explanatory variable number m in the stepwise regression process, i.e., $p_{k}=\frac{m}{n}$, *n* represents the total number of explanatory variables (*n* = 126, *m* = 25).

Step 3: Calculate the level of talent attraction. First, a priori *γ* is obtained based on formula (1) (2). Then, the corresponding explanatory variable set X* of *β*_*k*_≠0 is obtained according to *γ*_*k*_ = 1 and selected as the set of explanatory variables for the current stepwise regression. After the regression, the fitting coefficient β* corresponding to X* is obtained. The sampling is repeated 10,000 times (to ensure the convergence of the model). The mean value of the coefficients obtained from each regression is used as the final coefficient of the explanatory variable and the weight of each evaluation index. Then, the index value and weight are substituted into formula (3) to obtain the level of talent attraction in each province *M* as follows:

$$M=\beta_{0}+\beta_{1}x_{1}^{\prime }+\beta_{2}x_{2}^{\prime }+\cdots +\beta_{126}x_{126}^{\prime }$$ (3)

The index value and weight are substituted into formula (4) to obtain score *M*_*i*_(*i* = 1,2,3,4) of the talent attraction level under the four first-level indicators *M*_*i*_(*i* = 1,2,3,4) as follows:

$$\begin{array}{l} M_{1}=\beta_{1}x_{1}^{\prime }+\beta_{2}x_{2}^{\prime }+\cdots +\beta_{24}x_{24}^{\prime }\\ M_{2}=\beta_{25}x_{25}^{\prime }+\beta_{26}x_{26}^{\prime }+\cdots +\beta_{55}x_{55}^{\prime }\\ M_{3}=\beta_{56}x_{56}^{\prime }+\beta_{57}x_{57}^{\prime }+\cdots +\beta_{76}x_{76}^{\prime }\\ M_{4}=\beta_{77}x_{77}^{\prime }+\beta_{78}x_{78}^{\prime }+\cdots +\beta_{126}x_{126}^{\prime } \end{array}$$ (4)

where $x_{j}^{\prime }(j=1,2,\cdots ,126)$ represents a column vector comprising the observed values after the standardization of the *j*^*th*^ evaluation indicator, and *β*_*j*_(*j* = 1,2,⋯,126) is an indicator weight.

### Spatial autocorrelation model

#### Global spatial autocorrelation model

The Moran’s I index is used to calculate the degree of global autocorrelation [32], and then the spatial correlation and concentration of talent attraction levels are analyzed. The calculation formula of Moran’s I index is as follows:

$$I=\frac{n\sum_{i=1}^{n}\sum_{j\neq i}^{n}w_{ij}(m_{i}-\bar{m})(m_{j}-\bar{m})}{\sum_{i=1}^{n}\sum_{j\neq i}^{n}w_{ij}\sum_{i=1}^{n}{\left(x_{i}-\bar{x}\right)}^{2}}$$ (5)

Where, *n* is the number of regional units in the area, that is the total number of provinces (*n* = 31); *m*_*i*_,*m*_*j*_ are the talent attraction level of *i*,*j*, respectively; $\bar{m}$ is mean value; *w*_*ij*_ is distance weighd. If 0<*I*≤1, it indicates that the level of talent attraction in various regions is positively correlated; if −1≤*I*<0, there is a negative correlation; if *I* = 0, there is no spatial correlation.

For Moran's I index, standardized statistics *Z* are generally used to test the significance of spatial autocorrelation. Its calculation formula is as follows:

$$Z=\frac{I-E(I)}{\sqrt{VAR(I)}}$$ (6)

Where, *E*(*I*) and *VAR*(*I*) are the theoretical expectation and theoretical variance of *I*, respectively.

If *Z* is positive and significant, it indicates that there is a significant positive correlation, that is, similar observations tend to gather in space. If *Z* is negative and significant, it indicates that there is a significant negative correlation, that is, similar observed values tend to be dispersed. If *Z* value is 0, the observed values are distributed independently and randomly.

#### Local spatial autocorrelation model

After calculating the Geti-Ord G index, the distribution of cold-hot spot areas in the evolution process of talent attraction level is identified, and the formula is as follows:

$$G_{i}^{*}(d)=\frac{\sum_{j=1}^{n}w_{ij}m_{j}}{\sum_{i=1}^{n}m_{i}}$$ (7)

At the same time, there are corresponding standardized statistics *Z*(*i*)* for the index $G_{i}^{*}$:

$$Z{\left(i\right)}^{*}=\frac{G_{i}^{*}(d)-E\left[G_{i}^{*}\right]}{\sqrt{VAR\left[G_{i}^{*}\right]}}$$ (8)

Where, $E\left[G_{i}^{*}\right]$ and $\sqrt{VAR\left[G_{i}^{*}\right]}$ are the expectation and variance of $G_{i}^{*}$, respectively. If the value of *Z*(*i*)* is positive and significant, it indicates that the surrounding area of *i* is a cluster of high-value space (hot spot area). If the value is negative and significant, it indicates that the surrounding area of *i* is a low-value spatial agglomeration (cold spot area).

## Results

### Temporal characteristics of talent attraction

**Fig 1:**
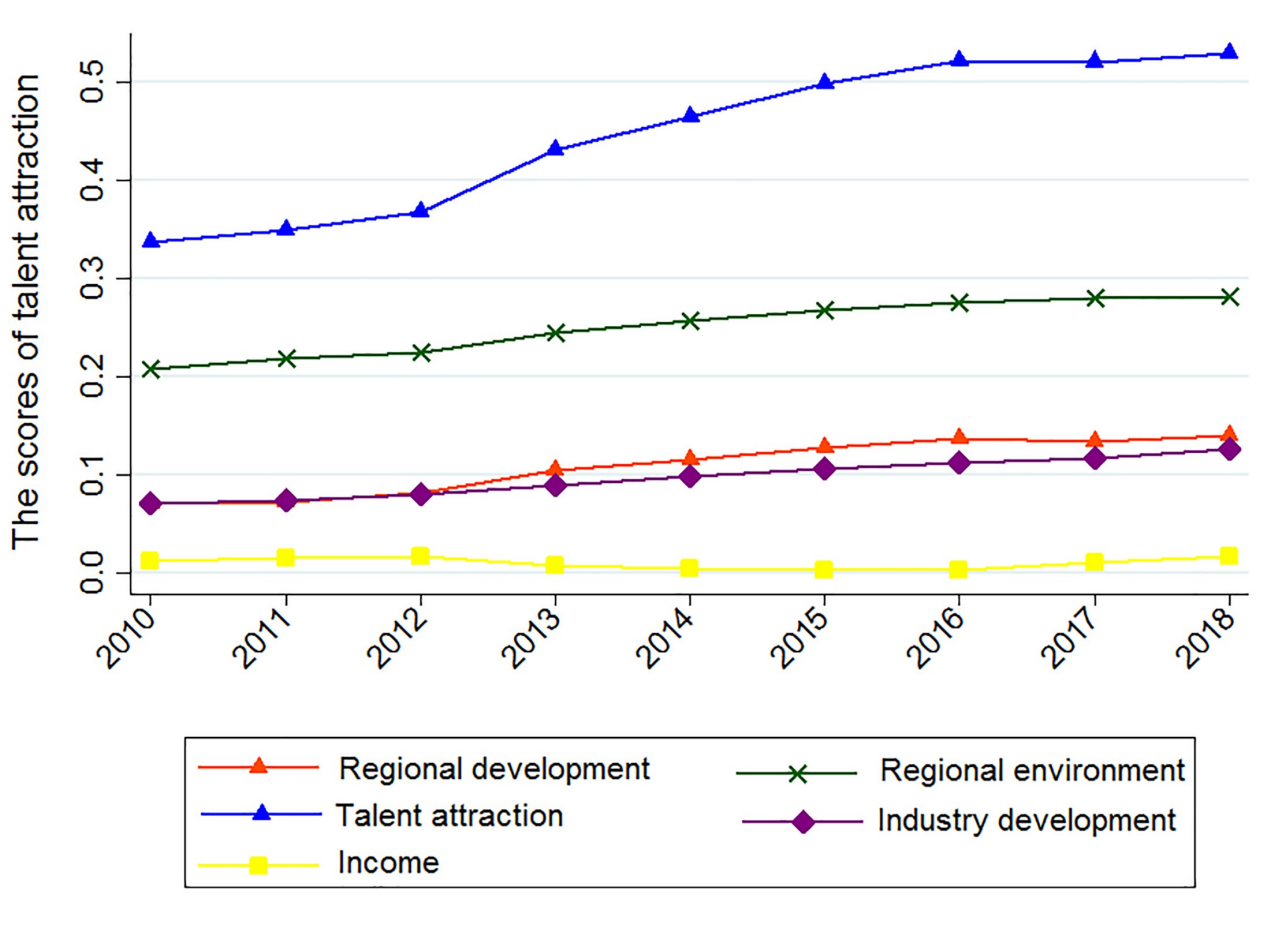
China's annual talent attraction and trends in various scores.

We apply formula (3) and formula (4) to calculate the talent attraction level in 31 provinces (cities) in China from 2010 to 2018, and their scores under the four levels of indicators are shown in Fig 1. Overall, from 2010 to 2018, the attractiveness level of China's provincial talents showed a steady upward trend with an average annual growth rate of 5.804%. On the one hand, this result shows that the competition for talents has become increasingly fierce in recent years. The talent introduction policies have promoted the flow of talents. Simultaneously, with the enhancement of China's comprehensive national strength, the introduction of overseas talents has also achieved considerable results. China is becoming more competitive in the international talent market. On the other hand, the results also show that due to the imbalance in development among different regions in China, the level of talent attraction highly differs across regions. The weak attraction of talents in many regions renders the overall talent attraction level relatively low. Governments and other institutions should continue to introduce reasonable and efficient talent introduction policies. As shown in Fig 1, from 2010–2012, the growth of talent attraction was slower. The economic crisis in 2010 caused China's economic growth rate to decrease from 11.9% in 2009 to 10% in 2010. In 2011, the growth rate further decreased to 9.5%, due to a decline in the economic growth rate, rise in the tide of corporate closures and unemployment rate and natural disasters impacting China’s economic and social development. Talents make more conservative choices, and the flow of talent during this period is slower. After 2012, with the recovery of China's economy, the growth of talent attraction has accelerated.

The scores of talent attraction under the four first-level indicators all show regional environment > regional development > industry development > income. This result shows that current talents pay more attention to the harmony of the social environment and livability of the natural environment. In particular, the cultural atmosphere, social environment, and open social and cultural atmosphere can enhance cities' cohesiveness and talents’ sense of belonging and security. The advantages of the social environment are mainly reflected in housing, medical care and education. Simultaneously, talents consider the current development level and future development potential of the region and the development of various industries. The level of regional economic development and the completeness of various industrial chains have become important factors influencing talent attraction. In addition, talents pay minimal attention to the income level, suggesting that talent introduction policies can provide adequate income security. Moreover, the income level gap across different regions is gradually narrowing, leading to fewer considerations for talents.

Judging from the trend of various scores, except for the score of income level, there is a downward trend in some years, and other factors are always increasing. The average annual growth rate of talent attraction under the four levels of comprehensive development, industry development, income and regional environment is 8.820%, 7.48%, 4.203% and 3.830%, respectively. The influence of the comprehensive development level on the attraction of talents increases the fastest, and the influence of the industry development level is also increasing rapidly. The rapid development of industry has promoted improvement in the regional comprehensive strength. Although the regional environment has the greatest impact on the level of talent attraction, the growth rate is slower. The income level has a minimal impact on the attractiveness of talents, and its growth rate is also slower.

### Spatial characteristics of talent attraction

To reflect the spatial distribution characteristics of talent attraction in various regions, according to the calculation results of the talent attraction level of provinces and cities in 2010–2018, the talent attraction level of each province and city is divided into six levels to explore the spatial distribution differences of talent attraction (Table 2). Overall, the level of talent attraction shows a clear spatial distribution difference, and the overall trend is “decreasing from the east to the west and weakening from coastal to inland areas”.

**Table 2:** Spatial variation in talent attraction in China from 2010 to 2018.

Economic zone	Talent attraction				Economic zone	Talent attraction				
Eastern coastal areas	Shanghai	Zhejiang	Jiangsu		Northern coastal areas	Beijing	Tianjin	Hebei	Shandong	
2010	3.52	0.41	0.45		2010	1.99	1.02	0.15	0.31	
2012	3.74	0.47	0.52		2012	2.21	1.05	0.18	0.37	
2015	5.59	0.61	0.73		2015	2.61	1.45	0.23	0.50	
2018	5.86	0.69	0.85		2018	2.74	1.43	0.24	0.54	
Southern coastal areas	Fujian	Hainan	Guangdong		The middle Yangtze River	Anhui	Jiangxi	Hubei	Hunan	
2010	0.19	0.16	0.39		2010	0.16	0.11	0.15	0.13	
2012	0.22	0.12	0.42		2012	0.19	0.12	0.16	0.15	
2015	0.29	0.17	0.56		2015	0.25	0.15	0.22	0.19	
2018	0.31	0.20	0.66		2018	0.26	0.16	0.26	0.22	
The middle Yellow River	Shanxi	Neimenggu	Henan	shaanxi	Southwest areas	Guangxi	Chongqing	Sichuan	Guizhou	Yunan
2010	0.12	0.01	0.22	0.10	2010	0.08	0.17	0.07	0.07	0.05
2012	0.14	0.02	0.26	0.11	2012	0.10	0.19	0.08	0.07	0.05
2015	0.15	0.02	0.35	0.14	2015	0.12	0.29	0.11	0.10	0.07
2018	0.15	0.02	0.38	0.16	2018	0.13	0.32	0.12	0.12	0.08
Northeastern area	Liaoning	Heilongjiang	Jilin		Northwest areas	Xizhang	Gansu	Qinghai	Ningxia	Xinjiang
2010	0.21	0.05	0.10		2010	0.00	0.02	0.00	0.04	0.01
2012	0.24	0.06	0.10		2012	0.00	0.02	0.00	0.06	0.01
2015	0.28	0.06	0.11		2015	0.00	0.02	0.00	0.06	0.01
2018	0.28	0.06	0.11		2018	0.00	0.02	0.00	0.06	0.01

At the provincial level, the provinces and cities with higher talent attraction levels mainly include Beijing, Shanghai, Tianjin, Guangdong, Jiangsu, Zhejiang and other economically developed provinces and cities. The level of talent attraction in economically backward regions, such as Xinjiang, Tibet, Qinghai, Gansu, Yunnan, and Guizhou, is relatively low. This result shows that the level and speed of economic development have a very important impact on talent selection. The attractiveness of talents in the Beijing, Shanghai and Guangdong regions has been at a high level despite the potential housing and living pressures. Talents are still willing to relocate to these developed cities, resulting in the faster renewal of talents in economically developed cities and a slower improvement in the talent structure of cities with relatively backward economies. From 2010 to 2018, the attractiveness of talents in Beijing, Tianjin and Shanghai was greater than 1.020 and gradually increased. The level of talent attraction in Guangdong, Zhejiang and Jiangsu increased from 0.386–0.452 to 0.661–0.848. These results show that cities that dominate the talent competition have a faster growth rate. Provinces with attractive talents between 0.214 and 0.350 increased from 3 in 2010 to 8 in 2018, while provinces between 0.014–0.095 decreased from 9 to 5; talent attraction in Tibet, Qinghai and Xinjiang has been below 0.009 and shows a decline. The above changes show that except for some provinces and cities, the attractiveness of talents is gradually increasing. However, due to the imbalance in inter-regional development and the inherent status of talent competition, this upward trend shows a gradual weakening trend from the east to the west, resulting in the phenomenon that "the strong are stronger and the weak are weaker".

From the perspective of the eight economic zones, there is a clear difference in each region. The eastern and northern coastal areas rely on their unique geographical advantages and well-established economic foundation to create conditions for talent introduction. Therefore, these areas are in a dominant position in the talent competition, and talent attraction in these regions is significantly higher than that in other regions, i.e., almost 10 times higher than that in northeast China. Talent attraction is relatively similar in the middle Yellow River and southwestern and northeastern regions with scores ranging from 0.121 to 0.159, representing an intermediate level in the country. Talent attraction in the northwestern region is relatively low. The level of talent attraction from 2010 to 2012 is as follows: “East Coastal > North Coast > Southern Coast > Middle Yangtze River > Northeast > Middle Yellow River > Southwest > Greater Northwest”. By 2018, the rank changed to "Eastern Coastal > Northern Coastal > Southern Coastal > Middle Yangtze river > Middle Yellow River > Southwest > Northeast > Northwest”. Compared to the northeast, a rapid increase in the level of talent attraction can be observed in the middle Yellow River and southwest regions because the middle Yellow River is close to the Yellow River Basin, and natural resources are abundant. Simultaneously, irrigation convenience promotes the development of characteristic agriculture, which drives economic growth. The tertiary industry in the southwest region is relatively developed, and the development of tourism enables an increasing number of potential settlers, thus promoting the influx of talents. In recent years, the trend of labor outflow in the northeast region has become increasingly obvious and an important factor restricting economic development in this region. As a result, the growth of talent attraction in northeast China has declined. In contrast, the attractiveness of talents in the northwest region has remained at a low level. Factors, such as poor environmental conditions and inconvenient transportation, have always limited development. The difficulty in introducing talents is also an important reason for the long-term slow development.

### Spatial correlation characteristics of talent attraction

To study the spatial agglomeration pattern and evolution rule related to the talent attraction levels of provinces and cities from 2010 to 2018, the global autocorrelation (Moran's) index is calculated by using the global autocorrelation method as shown in Table 3.

**Table 3:** Global autocorrelation of talent attraction levels in China during the 2010–2018 period.

Talent Attraction	2010	2012	2015	2018
Moran's I	0.256	0.261	0.273	0.275
Z(I)	5.218	5.270	5.609	5.651
P(I)	0.000	0.000	0.000	0.000

According to Table 3, the value of the global autocorrelation index from 2010 to 2018 is between 0.256–0.275, and the Z(I) value is relatively large, indicating that the talent attraction levels of the provinces and cities have a strong positive correlation in space, and the trend of spatial agglomeration is more obvious. From 2010 to 2018, the value of Moran′s I index continuously increases from 0.256 to 0.275, showing a gradually strengthened phenomenon of spatial agglomeration of talent attraction in China.

To further explore the talent attraction level agglomeration phenomenon and identify the concentration distribution, the local spatial autocorrelation method is used to calculate the Geti-Ord G index of various provinces and cities. Additionally, according to the natural breaking point method, the country is divided into a cold spot area, sub-cold spot area, hot spot area and sub-hot spot area. The map describes the talent attraction level in the cold-hot spot area of various provinces and cities as shown in Table 4.

**Table 4:** The cold-hot area of the talent attraction level trend in various provinces and cities.

Provinces(cities)	2010	2012	2015	2018
**Beijing**	2.456	2.486	2.401	2.358
**Shanghai**	2.299	2.362	2.264	2.259
**Tianjin**	2.201	2.218	2.156	2.107
**Hebei**	2.201	2.218	2.156	2.107
**Shandong**	2.174	2.222	2.136	2.114
**Jiangsu**	2.090	2.153	2.055	2.047
**Anhui**	1.973	2.036	1.957	1.973
**Henan**	1.849	1.903	1.841	1.828
**Zhejiang**	1.694	1.735	1.810	1.870
**Hubei**	1.627	1.694	1.633	1.679
**Fujian**	1.567	1.595	1.666	1.752
**Guangdong**	1.437	1.457	1.330	1.280
**Jiangxi**	1.297	1.336	1.427	1.514
**Neimenggu**	0.243	0.281	0.079	0.010
**Jilin**	-0.386	-0.424	-0.453	-0.524
**Shanxi**	-0.462	-0.414	-0.540	-0.576
**Heilongjiang**	-0.648	-0.674	-0.675	-0.706
**Shaanxi**	-0.674	-0.641	-0.748	-0.795
**Xinjiang**	-0.741	-0.759	-0.715	-0.732
**Hainan**	-0.855	-0.883	-0.863	-0.800
**Xizang**	-0.920	-0.942	-0.889	-0.910
**Yunnan**	-0.984	-1.021	-0.962	-0.946
**Qinghai**	-1.021	-1.046	-0.988	-1.012
**Gansu**	-1.091	-1.111	-1.056	-1.080
**Liaoning**	-1.139	-1.160	-1.124	-1.068
**Ningxia**	-1.190	-1.191	-1.134	-1.149
**Guangxi**	-1.315	-1.345	-1.295	-1.242
**Guizhou**	-1.354	-1.385	-1.326	-1.283
**Chongqing**	-1.580	-1.588	-1.531	-1.505
**Hunan**	-1.609	-1.597	-1.560	-1.484
**Sichuan**	-1.609	-1.634	-1.549	-1.562

Red indicates the hot spots area; Orange indicates the sub-hot spot area; Blue indicates the sub-cold area; Purple indicates the cold spot area.

As shown in Table 4, from 2010 to 2018, the distribution of cold-hot spot areas of talent attraction level is similar, showing a transition trend from the east to the west. In 2010 and 2012, the numbers of talent attraction level cold spot, sub-cold spot, sub-hot spot and hot spot areas are 12, 6, 6 and 7, respectively. In 2015, the numbers of these areas are 10, 8, 4 and 9. Compared with previous years, the numbers of cold spot and hot spot areas decreased in 2015, while the number of cold spot and hot spot areas increased. This result indicates that talent attraction in each city has improved to different degrees. The number of cold spot, sub-cold spot, sub-hot spot and hot spot areas changed to 11, 7, 4 and 9, respectively, in 2018, with minimal overall change, indicating that the talent attraction level in China from 2015 to 2018 presents a relatively stable agglomeration phenomenon. In addition, the number of hot spot and cold spot areas is greater, and these areas are concentrated in the eastern coastal zone and western region, respectively. These results show a very uneven level of talent attraction in different regions.

Specifically, Beijing, Tianjin, Shanghai, Jiangsu, Zhejiang and other provinces are hot spot areas belonging to an agglomeration area with a high talent attraction level. The spatial differentiation of the talent attraction levels shows that the condition reflects the phenomenon of "clustering" of the distribution of talents. The existence of hot spot areas can promote talent attraction in surrounding cities to some extent. Fujian, Guangzhou, Jiangxi, Hubei and other provinces belong to sub-hot spot areas, indicating the presence of agglomeration areas with higher talent attraction but a lower level than that of hot spot areas. Shanxi, Shaanxi, Jilin, Inner Mongolia and other places belong to sub-cold spot areas, and the measurement results of the talent attraction level also show a relatively low level of talent attraction in these cities. Most provinces, such as Xizang, Gansu, Qinghai, Yunnan, and Guizhou, and other western regions belong to cold spot areas, which have lower talent attraction levels. Regarding annual changes, the number of cold spot areas has decreased, demonstrating that the agglomeration phenomenon of low talent attraction levels is gradually weakening. The number of hot spot areas has increased but to a small extent. For some regions with low talent attraction levels, it is very difficult to quickly improve the level over a short time. Provinces and cities should consider the local conditions and capitalize on regional advantages as much as possible to compensate for the weakness in attracting talents. Concurrently, the government should formulate relevant policies for talent introduction such that more talents can help build and take root in the western region and promote reform and development in the western region.

## Analyzing the influencing factors of talent attraction

The talent attraction level of provincial cities is of great significance to talent employment, and a comprehensive evaluation index of regional talent attraction affects the level of talent attraction. Among them, the regional comprehensive development level, industry development level, income level and regional environment have different degrees of impact on talent attraction. Thus, this paper further analyzes the key influencing factors affecting the attractiveness of talent. First, according to the measurement model of the talent attraction level, the regression coefficients of each index and the 10 indicators with the greatest influence on the talent attraction level (i.e., the 10 indicators with regression coefficients of the largest absolute value) can be obtained and classified according to the secondary indicators. The level of talent attraction is mainly affected by the national economy, people's lives, tertiary industry development, medical care, education, transportation, etc.

**Fig 2:**
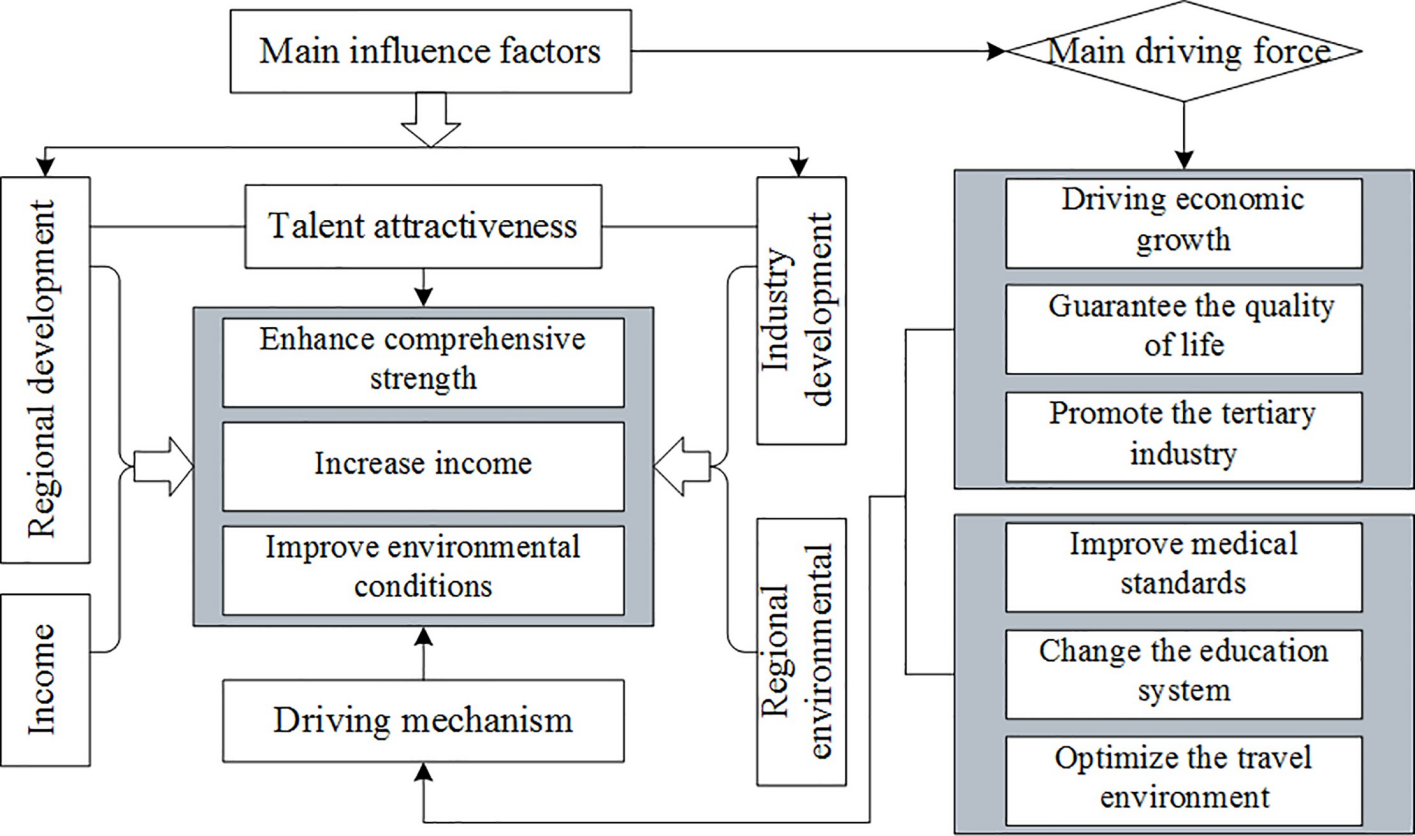
Factors influencing and mechanism driving talent attraction.

The overall framework of the factors influencing and mechanism driving talent attraction is shown in Fig 2. According to the regression coefficients of each influencing factor, the following can be observed.

1. Regional development level. The comprehensive regional development level has a significant impact on talent attraction, and the total wages of urban employees, gross regional product, proportion of urban population to the total population and other indicators also have a great impact. The regression coefficients are all above 0.428. The regional comprehensive development level mainly increases via economic growth and people's quality of life to improve the attractiveness of talent. For example, the economic development level and people's quality of life in Beijing and Shanghai are both relatively high as is talent attraction. However, Tibet, Qinghai and other areas show the opposite pattern. On the one hand, economic growth improves the regional GDP and loanable funds in banks; thus, enterprises can obtain sufficient funds to expand production, increasing the demand for talents. The more money available in the market, the more motivated talents are to start their own businesses, which can promote the influx of talents. In addition, economic growth leads to the movement of the demand and supply curve, increasing people's consumption and promoting economic growth, thus forming a virtuous circle. On the other hand, with the improvement in people's living standards, talents have increased requirements. Talents choose jobs not only for short-term stability but also to ensure long-term quality of life. Therefore, living standards have a strong impact on areas where talents are employed.
2. Industry development level. According to the industrial added value, added value index and fixed asset investment, the development level of each industry has a distinct influence on talent attraction. Notably, some industries in the tertiary industry of the talent attraction level have a great positive influence. For example, the value added index of the tertiary industry, the value added of the retail industry and the value added of the financial industry all have a regression coefficient greater than 0.301, indicating that the development of the tertiary industry plays a highly prominent role in promoting the attraction of regional talents. First, the development level of the tertiary industry generally reflects the development level of regions. Developed regions, such as Beijing, Shanghai and Guangzhou, have a higher development level of the tertiary industry. In particular, the rapid development of various service industries leads to a large number of jobs, creating conditions for the influx of talents. Second, the development of education, information transmission, computer, scientific research, technical services, geological survey and other industries in the tertiary industry cannot be separated from some highly educated and high-tech talents. The development of these industries affects the employment choices of many talents.
3. Income level. The income level of the industry partially reflects the development level of the industry. The coefficient of indicators, such as scientific research, average wage in wholesale and retail trade, are all above 0.646, demonstrating a strong positive correlation with the talent attraction level and further indicating that the development level of some industries in the tertiary industry has a great influence on the level of talent attraction. Although the income level in various regions is shrinking and the influence of income on the selection of talents is no longer dominant, currently, raising the wage level in some industries with a high demand for high-end talents is still a direct and effective method to attract talents. There is also a positive correlation between the average salary of urban employees and talent attraction (coefficient of 0.380), indicating that the higher the overall income level, the higher the talent attraction. A small number of industries have a relatively high negative correlation with the talent attraction level. For example, the construction industry (coefficient of -0.440) reflects a higher development level. High housing prices increase pressure to employ talents; thus, the average salary in the construction industry is negatively correlated with the attractiveness of urban talents.
4. The regional environmental level. The indicators of the regional environment are divided into the social and natural environment. The social environment mainly includes traffic, education, medical treatment, shopping, housing and public security. The natural environment mainly includes pollution and greening. Overall, the regional environment plays a role in attracting talents mainly through indexes related to medical treatment, education and transportation, such as the number of health technicians, ordinary colleges and universities and the total passenger volume of public transportation. The regression coefficients of these indicators all exceed 0.241. Improvement in the medical environment could help create a good social atmosphere in the region, thereby increasing the confidence of migrant workers and local residents in health care, effectively avoiding the brain drain and promoting the inflow of talents. Children's education is an important issue for talents to consider when choosing a career. Most talents choose to stay in a certain place not for their own sake but for their offspring to have a more comprehensive development platform and broader development space. Therefore, it is important for the reform of the education system to guarantee that children receive a good education and enjoy the same education rights as the local residents. Additionally, the convenience of transportation within a region is a major factor affecting the selection of talents. The convenience of travel is conducive to improving the efficiency of various work. In some cities with high population densities, tactics used to improve the convenience of travel have become an urgent problem to be solved. Moreover, in some areas with long-term unchanged traffic, traffic problems represent an important factor restricting development and improvement in talent attraction.

## Discussion

Compared with previous similar studies, our research contributes to the following three aspects. Firstly, we built a better indicator system, supplementing the influencing indicators drawn from across several decades, to create a set of 126 of them. Then, a sampling stepwise regression model was proposed to calculate the impacts of these influencing indicators on the attractiveness of regional talent. Compared with previous quantitative methods, such as the Analytic Hierarchy Process (AHP), Entropy Evaluation Method, or Principal Component Analysis (PCA), our model involves many more indicators because we believe that a city’s talent attractiveness comes from a lot of aspects rather than just a few. We therefore define a large set of influencing indicators to distinguish more accurately how these indicators reveal the positive or negative impacts on city attractiveness.

Secondly, our model assumes that most of the influencing indicators should be included in the model, even if each indicator captures only a small fraction of a city’s talent attractiveness. However, the indicators could be strongly correlated. Thus, we need to avoid multicollinearity between these independent variables when solving the model. To avoid the problem, we stochastically estimate a subset of our indicators in a stepwise regression process. The regression process is sufficient to ensure that the results converge. In this way, all the indicators that do affect a city’s talent attractiveness can be preserved. In summary, we solved the model by combining a spike-slab sampling process and stepwise regression based on the law of large numbers.

Thirdly, differently to similar studies that have often only focused on a single or limited regions or enterprises, we paid attention to the 31 provinces or province-level municipalities, as well as making a comparison among them across both the temporal and the spatial dimension. We analyzed the spatial and temporal distribution characteristics of talent attractiveness in the different regions of China. On the one hand, the talent attractiveness level of China's provinces shows a steady upward trend over time. On the other hand, there is significant spatial imbalance in and agglomeration of the overall level of talent attraction in different provinces. For example, the developed cities on the east and west coasts, such as Beijing and Shanghai, have retained a high level of talent attraction, while inland provinces such as Tibet and Qinghai have low levels over long periods.

In fact, our research comes to some similar conclusions to previous studies. For example, Li and Liu [7] found that the indicators of a city’s development status, per capita income and the output proportion of tertiary industry are all significant influencing indicators for talent attraction. In our research, we found that the indicators of GRP (Gross Regional Product), the average salary of employees and the value-added of the tertiary industry were positively correlated with a city’s talent attraction, verifying the previous conclusions mentioned above. Besides this, we found that the regional environment plays a role in attracting talent, mainly through indexes related to medical treatment, education and transportation, reflecting a similar view to Camille [33] and Anthony [34], who posited that building a beautiful modern city suitable for living well could create an environment that would give a city a comparative advantage regarding talent attraction. We have also produced some unpopular conclusions, however. For example, we found that talented individuals in China tend to choose jobs in cities with a developed service industry rather than primary industry. Meanwhile, compared to job income, the social environment, including aspects such as education, traffic and shopping services, often plays a much greater role in attracting talent to cities.

Since the economic development status tends to vary across different provinces and province-level municipalities, there is a variety of relevant policies on talent attraction in China. Talent in central China lags behind that in eastern China in terms of both stock and quality. For example, both Beijing and Shanghai are megacities in China that are highly talent attractive, and whose talent policies are more attractive due to providing much more money and opportunities [35]. However, the talent selection process in these cities is often based on much more rigorous tests of competence and experience. Thus, while the talent stock in these cities is increasing, the growth rate is only rising slowly. On the contrary, the talent attraction in provinces such as Jiangsu and Zhejiang is moderate [36]. These regions take full advantage of natural resources and geographical location, and their natural resources make them especially competitive in the tourism market. The talent policies in these places are also very attractive, while their talent selection process is much easier to succeed at. Consequently, a lot of talent is crowding into these regions, producing a sharp increase in both talent attraction and talent stock. As for the regions far away from the eastern coast, such as Gansu and Qinghai provinces, because of their poor economies and location, their talent attraction has remained relatively low for an extended period. Although they provide a convenient green channel [37] for talented people who are interested in working in western China (e.g. with high wages and housing), the lack of corresponding services and other aspects, such as the education of children and medical services, means these policies have little effect on talent attraction. These regions should therefore look to improve these aspects because people pay more attention to the regional medical level, education security and transportation convenience when choosing jobs.

## Conclusions

This paper examines the spatiotemporal dynamic pattern evolution of talent attraction in provinces and uses the Bayesian prior function and stepwise regression to construct a regression equation of the relationship between the number of employees and influencing factors and obtain the weight of each evaluation index. Then, the talent attraction level of each province is calculated. Finally, the degree of influence and mechanism of action of each index are determined. The conclusions are as follows.

According to the characteristics of the time series evolution, the level of talent attraction in China's provinces shows a steady rise with an average annual growth rate of 5.804%. Regarding the four primary indicators, the regional environment score is the highest, followed by the regional comprehensive and industrial development levels; the income score is the lowest. In addition, the influence of various factors on talent attraction has increased to different degrees. From the perspective of spatial differentiation, the overall level of talent attraction shows a decreasing trend from the east to the west. From 2010 to 2012, the average annual average was as follows: east coast > north coast > south coast > middle reaches of the Yangtze river > northeast > southwest > northwest; after 2012, the middle reaches of the Yellow River and the southwest region gradually surpassed the northeast region. Beijing, Shanghai and other developed cities have retained a high level of talent attraction, while Tibet and Qinghai have a low level with minimal change.

From the perspective of spatial correlation characteristics, the spatial agglomeration trend of talent attraction level in China is relatively obvious and gradually increases over the years. The numbers of hot spot and cold spot areas are both large and concentrated in the east and west coasts, respectively. The numbers of cold spot and sub-hot spot areas are decreasing, while the numbers of sub-cold spot and hot spot areas are increasing. Regarding the influence of various indicators on the talent attraction level, the regional economic development level, people's living standards and the development level of the tertiary industry all have a great impact on talent attraction. In addition, talents pay more attention to the regional medical level, education security and transportation convenience when choosing jobs.

According to the above analysis, the following steps are recommended to improve the level of talent attraction in Chinese provinces (cities) from the following aspects. (1)The large difference in the talent attraction level across different regions is mainly due to the unbalanced development of different regions, which restricts improvement in the overall development level. Under the guidance of the new development concept, all provinces (municipalities) should continue to intensify their efforts to develop the economy, deepen supply-side structural reform, promote reform in quality, efficiency and the driving force of economic development, and create conditions for talents. (2) Local governments should increase their investment in talent introduction by providing preferential housing policies and social security policies, focusing on the introduction of much-needed talents, and promoting the rational flow of talents. Regions with a backward comprehensive development level need to increase their investment and accelerate "talent manufacturing". To strengthen the training of local talents, some subsidies can be provided to college talents to prevent brain drain and compensate for the shortage of talent competition. (3)The relevant departments should optimize the living and social environments to increase the investment in education and medical treatment. Regarding the natural environment, we need to strengthen environmental protection, pursue green and sustainable development, and accelerate ecological progress.

## Supporting information

S1 Data (XLSX)

S1 Appendix (DOCX)
