# Supplementary material for: Understanding regional talent attraction and its influencing factors in China: From the perspective of spatiotemporal pattern evolution
Source: PLoS One. 2020 Jun 18;15(6):e0234856. doi: 10.1371/journal.pone.0234856 (PMC7302713; doi:10.1371/journal.pone.0234856)
Supplement: S1 Appendix — (DOCX) [file pone.0234856.s002.docx]

# S1 Appendix

**Table 1. Regional development indicators**

| **Secondary indicators** | **Tertiary indicators** |
| --- | --- |
| National economic accounts | Gross domestic product (hundred million) |
|  | GDP index(last year=100) |
|  | Total Retail Sales of Consumer Goods(hundred million) |
| Finance and investment | Local finance general budget revenue(hundred million) |
|  | Local finance general budget expenditure(hundred million) |
| Foreign economic trade | The use of foreign capital in the fixed assets of the whole society(hundred million) |
|  | The total amount of export at the place where the business unit is located(Thousand dollars) |
|  | The total amount of import at the place where the business unit is located(Thousand dollars) |
|  | Number of foreign-invested enterprises(household) |
|  | Total investment of foreign-invested enterprises(million dollars) |
| City overview | City population density(Person/square kilometer) |
|  | Comprehensive production capacity of water supply(thousands of cubic meters/day) |
|  | Road length(thousands of kilometers) |
|  | Urban water penetration rate(%) |
|  | Urban gas penetration rate(%) |
|  | Acceptance of domestic patent applications(item) |
| People's life | The proportion of urban population in the total population(%) |
|  | Total wages of employees of urban units(hundred million) |
|  | The number of registered unemployed in urban areas(ten thousand people) |
|  | The registered urban unemployment rate (%) |
|  | Public transport is available for every 10,000 people |
| Social security | On-the-job worker attends endowment insurance number(ten thousand people) |
|  | Basic endowment insurance expenditure income ratio (%) |
|  | Basic medical insurance for urban workers by the end of the year(ten thousand people) |

**Table 2. Industry development indicators**

| **Secondary indicators** | **Tertiary indicators** |
| --- | --- |
| Total social investment in fixed assets | Fixed assets investment in agriculture, forestry, animal husbandry, and fishery(hundred million) |
|  | The whole society fixed assets investment of mining industry(hundred million) |
|  | Total Investment in Fixed Assets of Manufacturing industry(hundred million) |
|  | Total Investment in Fixed Assets of electricity, gas and water production and supply industry(hundred million) |
|  | Total Investment in Fixed Assets of construction industry(hundred million) |
|  | Total Investment in Fixed Assets of transportation, warehousing and postal services(hundred million) |
|  | Total Investment in Fixed Assets of information transmission, computer services and software(hundred million) |
|  | Total Investment in Fixed Assets of wholesale and retail(hundred million) |
|  | Total Investment in Fixed Assets of accommodation and catering(hundred million) |
|  | Total Investment in Fixed Assets of financial sector(hundred million) |
|  | Total Investment in Fixed Assets of real estate industry(hundred million) |
|  | Total Investment in Fixed Assets of leasing and business services(hundred million) |
|  | Total Investment in Fixed Assets of scientific research, technical services and geological survey(hundred million) |
|  | Total Investment in Fixed Assets of water conservancy, environment and public facilities management(hundred million) |
|  | Total Investment in Fixed Assets of residential services and other services(hundred million) |
|  | Total Investment in Fixed Assets of education(hundred million) |
|  | Total Investment in Fixed Assets of health, social security and social welfare(hundred million) |
|  | Total Investment in Fixed Assets of culture, sports and entertainment(hundred million) |
|  | Total Investment in Fixed Assets of public administration and social organization(hundred million) |
| Added value in 19 industries | agriculture, forestry, animal husbandry, and fishery value added (hundred million) |
|  | industry value added(hundred million) |
|  | construction industry value added(hundred million) |
|  | wholesale and retail value added(hundred million) |
|  | transportation, warehousing and postal services value added(hundred million) |
|  | accommodation and catering value added(hundred million) |
|  | financial sector value added(hundred million) |
|  | real estate industry value added(hundred million) |
|  | other industries value added |
| Three major industrial value added indexes | the first industry value added index |
|  | the second industry value added index |
|  | the third industry value added index |

**Table 3. Income indicators**

| **Secondary indicators** | **Tertiary indicators** |
| --- | --- |
| The average salary of employees in 19 industries | Urban units average salary of employee in agriculture, forestry, animal husbandry, and fishery(yuan) |
|  | Urban units average salary of employee in mining industry(yuan) |
|  | Urban units average salary of employee in manufacturing(yuan) |
|  | Urban units average salary of employee in electricity, gas and water production and supply industry(yuan) |
|  | Urban units average salary of employee in construction industry(yuan) |
|  | Urban units average salary of employee in transportation, warehousing and postal services(yuan) |
|  | Urban units average salary of employee in information transmission, computer services and software(yuan) |
|  | Urban units average salary of employee in wholesale and retail(yuan) |
|  | Urban units average salary of employee in accommodation and catering(yuan) |
|  | Urban units average salary of employee in financial sector(yuan) |
|  | Urban units average salary of employee in real estate industry(yuan) |
|  | Urban units average salary of employee in leasing and business services(yuan) |
|  | Urban units average salary of employee in scientific research, technical services and geological survey(yuan) |
|  | Urban units average salary of employee in water conservancy, environment and public facilities management(yuan) |
|  | Urban units average salary of employee in residential services and other services(yuan) |
|  | Urban units average salary of employee in education(yuan) |
|  | Urban units average salary of employee in health, social security and social services(yuan) |
|  | Urban units average salary of employee in culture, sports and entertainment(yuan) |
|  | Urban units average salary of employee in public administration and social organization(yuan) |
| The average salary of all employees | Urban units average salary of employee(yuan) |
|  | Average salary of employees on the job in urban units (yuan) |

**Table 4. Regional environment indicators**

| **Secondary indicators** | **Tertiary indicators** |
| --- | --- |
| Traffic and security | Number of public transport vehicles in operation |
|  | Number of buses and trolleys in operation |
|  | Total length of track line(km) |
|  | Total public transport passenger traffic(ten thousand) |
|  | Bus and tram passenger traffic(ten thousand) |
|  | Taxi |
|  | The total number of traffic accidents |
| Greening and pollution | Urban green area(hectares) |
|  | Green area of park(hectares) |
|  | The number in the park |
|  | The park area(hectares) |
|  | Green coverage rate of built-up area(%) |
|  | Total wastewater discharge(ten thousand tons) |
|  | Chemical oxygen demand discharge(ten thousand tons) |
|  | Ammonia nitrogen emission(ten thousand tons) |
|  | Sulfur dioxide emission(ton) |
|  | The volume of household garbage(ten thousand tons) |
|  | Forest land area(hectares) |
|  | Forest area(hectares) |
|  | Plantation area(hectares) |
|  | Forest coverage(%) |
|  | Forest stock(thousands of cubic meters) |
|  | Total afforestation area(thousands of hectares) |
|  | Investment in industrial pollution control has been completed(ten thousand yuan) |
|  | Investment in waste-water treatment project has been completed(ten thousand yuan) |
| Education and health care | Number of ordinary colleges and universities |
|  | The number of students in ordinary institutions of higher learning(ten thousand) |
|  | The total number of faculty and staff in ordinary institutions of higher learning(ten thousand) |
|  | The number of students in secondary vocational schools(ten thousand) |
|  | The number of ordinary high schools |
|  | The number of students in an ordinary high school(ten thousand) |
|  | Number of staff in ordinary high schools(ten thousand) |
|  | The number of ordinary primary and secondary schools |
|  | The number of students in an ordinary primary school(ten thousand) |
|  | Number of full-time teachers in ordinary primary schools(ten thousand) |
|  | Number of medical and health institutions |
|  | Number of health workers(ten thousand) |
|  | Number of health technicians(ten thousand) |
|  | Number of practicing (assistant) doctors(ten thousand) |
|  | Number of registered nurses(ten thousand) |
| Housing and shopping | Consumer price index(last year=100) |
|  | Price index of goods sold(last year=100) |
|  | Household consumption level(yuan) |
|  | Consumer price index(last year=100) |
|  | Floor area completed(million square meters) |
|  | Construction area of residential buildings(million square meters) |
|  | Completed area of residential buildings(million square meters) |
|  | Number of real estate development enterprises |
|  | The number of employees in real estate development enterprises |
|  | Residential housing sales(hundred million) |
